# Chlorido(1*H*-imidazole-κ*N* ^3^)bis­(triphenyl­phosphane-κ*P*)copper(I)

**DOI:** 10.1107/S1600536811017752

**Published:** 2011-05-20

**Authors:** Moayad Hossaini Sadr, Reza Kia, Behzad Soltani

**Affiliations:** a Department of Chemistry, Azarbaijan University of Tarbiat Moallem, Tabriz, Iran; b X-ray Crystallography Laboratory, Plasma Physics Research Center, Science and Research Branch, Islamic Azad University, Tehran, Iran

## Abstract

In the title complex, [CuCl(C_3_H_4_N_2_)(C_18_H_15_P)_2_], the coordination geometry around Cu^I^ is distorted tetra­hedral formed by two triphenyl­phosphane ligands, an imidazole ligand and a chloride group. An intra­molecular C—H⋯Cl inter­action occurs. The crystal packing is stabilized by inter­molecular N—H⋯Cl hydrogen bonds, which form an extended chain parallel to [010].

## Related literature

For standard bond lengths, see: Allen *et al.* (1987 ▶). For background to the use of imidazole-derived ligands in coordination chemistry, see, for example: Trofimenko (1993 ▶); Sadimenko & Basson (1996 ▶); Pettinari (2001 ▶); Hossaini Sadr *et al.* (2005 ▶); Kitajima (1992 ▶); Kitajima *et al.* (1989 ▶).
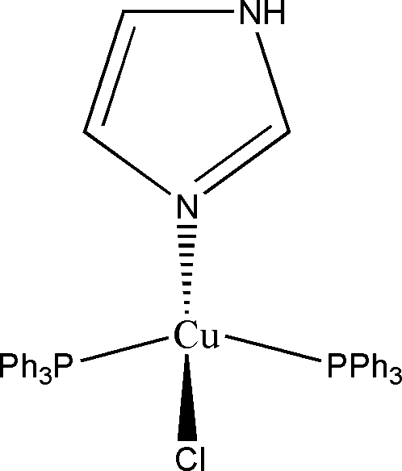


## Experimental

### 

#### Crystal data

[CuCl(C_3_H_4_N_2_)(C_18_H_15_P)_2_]
*M* *_r_* = 691.61
Monoclinic, 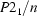

*a* = 13.674 (5) Å
*b* = 12.407 (5) Å
*c* = 20.353 (5) Å
β = 98.956 (5)°
*V* = 3411 (2) Å^3^
*Z* = 4
Mo *K*α radiation
μ = 0.84 mm^−1^
*T* = 296 K
0.42 × 0.41 × 0.35 mm

#### Data collection

Stoe IPDS 2T Image Plate diffractometer
Absorption correction: multi-scan (*MULABS* in *PLATON*; Blessing, 1995 ▶; Spek, 2009 ▶) *T*_min_ = 0.879, *T*_max_ = 1.000
24488 measured reflections
9190 independent reflections
6720 reflections with *I* > 2σ(*I*)
*R*_int_ = 0.053

#### Refinement

*R*[*F* ^2^ > 2σ(*F* ^2^)] = 0.046
*wR*(*F* ^2^) = 0.112
*S* = 1.02
9190 reflections
409 parameters
H atoms treated by a mixture of independent and constrained refinement
Δρ_max_ = 0.42 e Å^−3^
Δρ_min_ = −0.58 e Å^−3^

### 

Data collection: *X-AREA* (Stoe & Cie, 2009 ▶); cell refinement: *X-AREA*; data reduction: *X-AREA*; program(s) used to solve structure: *SHELXTL* (Sheldrick, 2008 ▶); program(s) used to refine structure: *SHELXTL*; molecular graphics: *SHELXTL*; software used to prepare material for publication: *SHELXTL* and *PLATON* (Spek, 2009 ▶).

## Supplementary Material

Crystal structure: contains datablocks global, I. DOI: 10.1107/S1600536811017752/jh2287sup1.cif

Structure factors: contains datablocks I. DOI: 10.1107/S1600536811017752/jh2287Isup2.hkl

Additional supplementary materials:  crystallographic information; 3D view; checkCIF report

## Figures and Tables

**Table 1:** Hydrogen-bond geometry (Å, °)

*D*—H⋯*A*	*D*—H	H⋯*A*	*D*⋯*A*	*D*—H⋯*A*
N2—H2⋯Cl1^i^	0.80 (4)	2.34 (4)	3.127 (3)	171 (3)
C5—H5*A*⋯Cl1	0.93	2.78	3.663 (4)	160

Symmetry code: (i) 
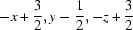
.

**Fig. 1.**
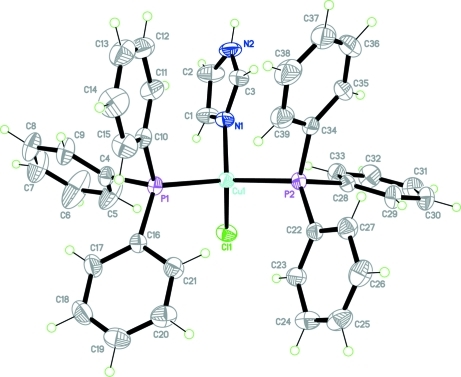
The ORTEP plot of the title compound, showing 40% probability displacement ellipsoids and the atomic numbering.

**Fig. 2.**
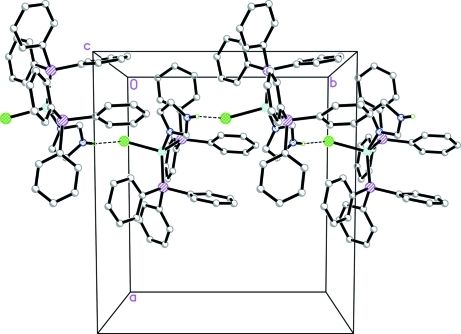
The packing diagram of the title compound viewed down the c-axis showing an extended chain along the b-axis. The dashed lines show the hyrogen bonding interactions.

## supplementary crystallographic information

### Comment

The chemistry of copper complexes with nitrogen-containing ligands especially pyrazole and imidazole derived ligands has been attracting continuous attention due to their rich coordination properties (Trofimenko, 1993; Sadimenko *et al.*, 1996) and show good models of the active sites of copper proteins (Pettinari, 2001; Hossaini Sadr *et al.*, 2005; Kitajima, 1992; Kitajima *et al.*, 1989).

The asymmetric unit of the title complex, Fig. 1, comprises one molecule of the complex. The bond lengths (Allen, *et al.*, 1987) and angles are within the normal ranges. The geometry around Cu^I^ is that of distorted tetrahedral which is coordinated by two triphenylphosphanes, imidazole and chloro groups. The crystal packing is stabilized by the intermolecular N—H···Cl hydrogen bonds (Table 2) which makes an extended chain along the [0 1 0] direction (Fig. 2).

### Experimental

PPh_3_ (2 mmol, 0.53 g) was added to a solution of CuCl (1 mmol, 0.09 g) and imidazole (2 mmol, 0.07 g) in dry CH_3_OH/CH_3_CN (1:1) (30 ml) and stirred for 12 h under N_2_ atmosphere. The filtrate of the resulting mixture was left to evaporate slowly at ambient temperature. Single crystals suitable for X-ray diffraction analysis were obtained after 4 days.

### Refinement

All hydrogen atoms were positioned geometrically with C–H = 0.93 Å and included in a riding model approximation with *U*_iso_ (H) = 1.2 *U*_eq_ (C), except the N-bound H atom which was located from the difference Fourier map and constrained to refine with the parent atom with *U*_iso_ (H) = 1.2 *U*_eq_ (N).

### Crystal data

**Table:**

[CuCl(C_3_H_4_N_2_)(C_18_H_15_P)_2_]	*F*(000) = 1432
*M**_r_* = 691.61	*D*_x_ = 1.347 Mg m^−^^3^
Monoclinic, *P*2_1_/*n*	Mo *K*α radiation, λ = 0.71069 Å
Hall symbol: -P 2yn	Cell parameters from 5052 reflections
*a* = 13.674 (5) Å	θ = 1.9–29.5°
*b* = 12.407 (5) Å	µ = 0.84 mm^−^^1^
*c* = 20.353 (5) Å	*T* = 296 K
β = 98.956 (5)°	Block, pale-yellow
*V* = 3411 (2) Å^3^	0.42 × 0.41 × 0.35 mm
*Z* = 4	

### Data collection

**Table:**

Stoe IPDS 2T Image Plate diffractometer	9190 independent reflections
Radiation source: fine-focus sealed tube	6720 reflections with *I* > 2σ(*I*)
graphite	*R*_int_ = 0.053
Detector resolution: 0.15 mm pixels mm^-1^	θ_max_ = 29.2°, θ_min_ = 1.7°
ω scans	*h* = −18→18
Absorption correction: multi-scan (*MULABS* in *PLATON*; Blessing, 1995; Spek, 2009)	*k* = −16→15
*T*_min_ = 0.879, *T*_max_ = 1.000	*l* = −27→27
24488 measured reflections	

### Refinement

**Table:**

Refinement on *F*^2^	Primary atom site location: structure-invariant direct methods
Least-squares matrix: full	Secondary atom site location: difference Fourier map
*R*[*F*^2^ > 2σ(*F*^2^)] = 0.046	Hydrogen site location: inferred from neighbouring sites
*wR*(*F*^2^) = 0.112	H atoms treated by a mixture of independent and constrained refinement
*S* = 1.02	*w* = 1/[σ^2^(*F*_o_^2^) + (0.0542*P*)^2^ + 0.4442*P*] where *P* = (*F*_o_^2^ + 2*F*_c_^2^)/3
9190 reflections	(Δ/σ)_max_ = 0.002
409 parameters	Δρ_max_ = 0.42 e Å^−^^3^
0 restraints	Δρ_min_ = −0.58 e Å^−^^3^

### Special details

**Table:**

Geometry. All e.s.d.'s (except the e.s.d. in the dihedral angle between two l.s. planes) are estimated using the full covariance matrix. The cell e.s.d.'s are taken into account individually in the estimation of e.s.d.'s in distances, angles and torsion angles; correlations between e.s.d.'s in cell parameters are only used when they are defined by crystal symmetry. An approximate (isotropic) treatment of cell e.s.d.'s is used for estimating e.s.d.'s involving l.s. planes.
Refinement. Refinement of *F*^2^ against ALL reflections. The weighted *R*-factor *wR* and goodness of fit *S* are based on *F*^2^, conventional *R*-factors *R* are based on *F*, with *F* set to zero for negative *F*^2^. The threshold expression of *F*^2^ > σ(*F*^2^) is used only for calculating *R*-factors(gt) *etc*. and is not relevant to the choice of reflections for refinement. *R*-factors based on *F*^2^ are statistically about twice as large as those based on *F*, and *R*- factors based on ALL data will be even larger.

### Fractional atomic coordinates and isotropic or equivalent isotropic displacement parameters (Å^2^)

**Table:**

	*x*	*y*	*z*	*U*_iso_*/*U*_eq_	
Cu1	0.841807 (18)	0.32155 (2)	0.629310 (13)	0.03820 (8)	
Cl1	0.79523 (5)	0.50608 (5)	0.61438 (3)	0.05424 (16)	
P1	0.78372 (4)	0.23056 (5)	0.53392 (3)	0.03740 (12)	
P2	1.00039 (4)	0.31129 (5)	0.68142 (3)	0.03835 (12)	
N1	0.75543 (15)	0.27483 (17)	0.69960 (10)	0.0480 (5)	
N2	0.6971 (2)	0.1876 (2)	0.77835 (12)	0.0645 (7)	
H2	0.692 (2)	0.141 (3)	0.8046 (17)	0.077*	
C1	0.6682 (2)	0.3194 (2)	0.70903 (15)	0.0613 (7)	
H1A	0.6384	0.3783	0.6858	0.074*	
C2	0.6310 (3)	0.2653 (3)	0.75720 (18)	0.0791 (10)	
H2A	0.5716	0.2790	0.7726	0.095*	
C3	0.7705 (2)	0.1959 (2)	0.74322 (13)	0.0551 (6)	
H3A	0.8260	0.1515	0.7487	0.066*	
C4	0.64932 (15)	0.2413 (2)	0.50960 (12)	0.0442 (5)	
C5	0.6049 (2)	0.3378 (3)	0.5176 (2)	0.0946 (14)	
H5A	0.6422	0.3953	0.5372	0.114*	
C6	0.5037 (3)	0.3503 (3)	0.4965 (3)	0.139 (2)	
H6A	0.4747	0.4174	0.5002	0.167*	
C7	0.4468 (2)	0.2669 (3)	0.4707 (2)	0.0949 (13)	
H7A	0.3791	0.2761	0.4571	0.114*	
C8	0.4895 (2)	0.1700 (3)	0.4650 (2)	0.0797 (10)	
H8A	0.4508	0.1116	0.4484	0.096*	
C9	0.59015 (19)	0.1571 (3)	0.48383 (17)	0.0677 (8)	
H9A	0.6186	0.0900	0.4790	0.081*	
C10	0.80015 (15)	0.08445 (19)	0.54037 (12)	0.0437 (5)	
C11	0.7684 (2)	0.0338 (2)	0.59392 (16)	0.0611 (7)	
H11A	0.7432	0.0753	0.6255	0.073*	
C12	0.7731 (2)	−0.0765 (2)	0.60157 (18)	0.0713 (8)	
H12A	0.7500	−0.1090	0.6374	0.086*	
C13	0.8120 (2)	−0.1378 (3)	0.5563 (2)	0.0774 (9)	
H13A	0.8155	−0.2123	0.5612	0.093*	
C14	0.8456 (3)	−0.0897 (3)	0.5040 (2)	0.0855 (10)	
H14A	0.8725	−0.1317	0.4735	0.103*	
C15	0.8403 (2)	0.0216 (2)	0.49573 (16)	0.0667 (8)	
H15A	0.8639	0.0535	0.4599	0.080*	
C16	0.83088 (15)	0.26438 (18)	0.45733 (11)	0.0408 (5)	
C17	0.7717 (2)	0.2776 (3)	0.39636 (13)	0.0675 (8)	
H17A	0.7040	0.2651	0.3924	0.081*	
C18	0.8118 (2)	0.3093 (3)	0.34108 (15)	0.0787 (10)	
H18A	0.7706	0.3187	0.3006	0.094*	
C19	0.9107 (2)	0.3267 (2)	0.34536 (15)	0.0645 (7)	
H19A	0.9371	0.3475	0.3079	0.077*	
C20	0.9710 (2)	0.3138 (2)	0.40473 (16)	0.0634 (7)	
H20A	1.0387	0.3260	0.4078	0.076*	
C21	0.93172 (17)	0.2824 (2)	0.46077 (13)	0.0519 (6)	
H21A	0.9735	0.2733	0.5010	0.062*	
C22	1.09967 (15)	0.33598 (18)	0.63312 (11)	0.0399 (5)	
C23	1.08348 (18)	0.4148 (2)	0.58503 (12)	0.0495 (5)	
H23A	1.0228	0.4502	0.5778	0.059*	
C24	1.1553 (2)	0.4422 (2)	0.54744 (15)	0.0617 (7)	
H24A	1.1429	0.4956	0.5151	0.074*	
C25	1.2447 (2)	0.3910 (2)	0.55775 (15)	0.0637 (7)	
H25A	1.2931	0.4091	0.5322	0.076*	
C26	1.26349 (19)	0.3128 (3)	0.60565 (16)	0.0650 (8)	
H26A	1.3248	0.2787	0.6127	0.078*	
C27	1.19130 (18)	0.2841 (2)	0.64382 (14)	0.0533 (6)	
H27A	1.2041	0.2308	0.6762	0.064*	
C28	1.03301 (17)	0.40460 (19)	0.75114 (11)	0.0443 (5)	
C29	1.13038 (19)	0.4304 (2)	0.77678 (14)	0.0593 (7)	
H29A	1.1820	0.3988	0.7588	0.071*	
C30	1.1511 (2)	0.5028 (3)	0.82877 (15)	0.0708 (8)	
H30A	1.2165	0.5187	0.8460	0.085*	
C31	1.0763 (3)	0.5510 (3)	0.85505 (15)	0.0723 (9)	
H31A	1.0907	0.5991	0.8903	0.087*	
C32	0.9796 (3)	0.5282 (3)	0.82932 (16)	0.0743 (9)	
H32A	0.9287	0.5618	0.8468	0.089*	
C33	0.9575 (2)	0.4556 (2)	0.77755 (13)	0.0593 (7)	
H33A	0.8918	0.4408	0.7604	0.071*	
C34	1.02986 (16)	0.1780 (2)	0.71692 (13)	0.0484 (5)	
C35	1.0459 (2)	0.1579 (3)	0.78493 (16)	0.0744 (9)	
H35A	1.0439	0.2138	0.8152	0.089*	
C36	1.0649 (3)	0.0526 (4)	0.8071 (2)	0.1134 (17)	
H36A	1.0757	0.0383	0.8525	0.136*	
C37	1.0679 (3)	−0.0304 (3)	0.7627 (3)	0.1120 (17)	
H37A	1.0827	−0.0999	0.7784	0.134*	
C38	1.0494 (3)	−0.0118 (3)	0.6959 (2)	0.0887 (11)	
H38A	1.0499	−0.0684	0.6660	0.106*	
C39	1.0300 (2)	0.0912 (2)	0.67346 (17)	0.0652 (7)	
H39A	1.0166	0.1036	0.6279	0.078*	

### Atomic displacement parameters (Å^2^)

**Table:**

	*U*^11^	*U*^22^	*U*^33^	*U*^12^	*U*^13^	*U*^23^
Cu1	0.03717 (13)	0.04272 (15)	0.03490 (14)	−0.00243 (11)	0.00620 (10)	−0.00078 (12)
Cl1	0.0774 (4)	0.0389 (3)	0.0486 (3)	0.0017 (3)	0.0166 (3)	−0.0012 (3)
P1	0.0326 (2)	0.0410 (3)	0.0380 (3)	−0.0004 (2)	0.0035 (2)	−0.0058 (2)
P2	0.0369 (3)	0.0426 (3)	0.0343 (3)	−0.0024 (2)	0.0017 (2)	0.0001 (2)
N1	0.0501 (10)	0.0520 (12)	0.0441 (10)	−0.0047 (9)	0.0144 (8)	0.0072 (9)
N2	0.0835 (17)	0.0596 (15)	0.0553 (14)	−0.0193 (13)	0.0257 (12)	0.0128 (12)
C1	0.0656 (16)	0.0573 (16)	0.0669 (17)	0.0049 (13)	0.0290 (14)	0.0147 (14)
C2	0.083 (2)	0.077 (2)	0.089 (2)	0.0050 (17)	0.0530 (19)	0.0175 (19)
C3	0.0586 (14)	0.0569 (15)	0.0504 (14)	−0.0049 (12)	0.0105 (11)	0.0117 (12)
C4	0.0331 (10)	0.0519 (13)	0.0474 (12)	−0.0006 (9)	0.0060 (9)	−0.0091 (11)
C5	0.0411 (14)	0.0577 (19)	0.179 (4)	0.0039 (12)	−0.0005 (19)	−0.032 (2)
C6	0.0459 (17)	0.081 (3)	0.280 (7)	0.0192 (17)	−0.004 (3)	−0.054 (4)
C7	0.0331 (13)	0.107 (3)	0.141 (4)	0.0036 (15)	0.0012 (17)	−0.040 (3)
C8	0.0421 (13)	0.088 (2)	0.104 (3)	−0.0121 (14)	−0.0022 (15)	−0.029 (2)
C9	0.0454 (13)	0.0660 (18)	0.087 (2)	0.0011 (12)	−0.0038 (13)	−0.0250 (16)
C10	0.0361 (10)	0.0427 (12)	0.0501 (13)	0.0011 (9)	−0.0002 (9)	−0.0070 (10)
C11	0.0649 (16)	0.0502 (15)	0.0710 (18)	−0.0048 (12)	0.0199 (14)	−0.0026 (14)
C12	0.0708 (18)	0.0534 (17)	0.089 (2)	−0.0063 (14)	0.0104 (16)	0.0109 (16)
C13	0.0659 (18)	0.0459 (16)	0.116 (3)	0.0061 (13)	−0.0002 (18)	−0.0003 (18)
C14	0.096 (2)	0.0576 (19)	0.106 (3)	0.0208 (17)	0.026 (2)	−0.0171 (19)
C15	0.0796 (19)	0.0543 (16)	0.0686 (19)	0.0126 (14)	0.0187 (15)	−0.0082 (14)
C16	0.0374 (10)	0.0439 (12)	0.0408 (11)	0.0020 (9)	0.0048 (8)	−0.0045 (10)
C17	0.0475 (14)	0.108 (2)	0.0440 (14)	−0.0136 (15)	−0.0012 (11)	0.0036 (16)
C18	0.0737 (19)	0.120 (3)	0.0405 (14)	−0.0134 (19)	0.0038 (13)	0.0014 (17)
C19	0.0763 (19)	0.0666 (18)	0.0568 (16)	0.0084 (15)	0.0296 (14)	0.0046 (14)
C20	0.0471 (13)	0.0696 (18)	0.079 (2)	0.0083 (12)	0.0257 (13)	0.0076 (16)
C21	0.0375 (11)	0.0632 (15)	0.0545 (14)	0.0064 (10)	0.0055 (10)	0.0048 (12)
C22	0.0396 (10)	0.0418 (12)	0.0374 (11)	−0.0018 (8)	0.0026 (8)	−0.0023 (9)
C23	0.0491 (12)	0.0506 (14)	0.0495 (13)	0.0009 (10)	0.0096 (10)	0.0051 (11)
C24	0.0745 (18)	0.0568 (16)	0.0589 (16)	−0.0055 (14)	0.0259 (14)	0.0065 (13)
C25	0.0661 (16)	0.0640 (17)	0.0680 (18)	−0.0150 (14)	0.0329 (14)	−0.0115 (15)
C26	0.0436 (13)	0.0723 (19)	0.082 (2)	0.0039 (12)	0.0178 (13)	−0.0128 (17)
C27	0.0457 (12)	0.0578 (15)	0.0563 (15)	0.0030 (11)	0.0079 (11)	−0.0002 (12)
C28	0.0492 (12)	0.0478 (13)	0.0345 (11)	−0.0056 (10)	0.0019 (9)	0.0005 (10)
C29	0.0520 (14)	0.0678 (18)	0.0548 (15)	−0.0040 (12)	−0.0024 (11)	−0.0105 (13)
C30	0.0719 (18)	0.078 (2)	0.0575 (17)	−0.0211 (16)	−0.0073 (14)	−0.0140 (16)
C31	0.101 (2)	0.0676 (19)	0.0473 (16)	−0.0198 (17)	0.0103 (16)	−0.0161 (14)
C32	0.087 (2)	0.080 (2)	0.0597 (18)	−0.0072 (17)	0.0245 (16)	−0.0227 (16)
C33	0.0596 (15)	0.0727 (18)	0.0467 (14)	−0.0055 (13)	0.0118 (12)	−0.0107 (13)
C34	0.0382 (11)	0.0486 (13)	0.0548 (14)	−0.0080 (10)	−0.0037 (10)	0.0095 (12)
C35	0.086 (2)	0.069 (2)	0.0578 (17)	−0.0182 (16)	−0.0219 (15)	0.0199 (15)
C36	0.139 (4)	0.086 (3)	0.093 (3)	−0.034 (3)	−0.049 (3)	0.044 (2)
C37	0.107 (3)	0.058 (2)	0.150 (4)	−0.018 (2)	−0.045 (3)	0.039 (3)
C38	0.085 (2)	0.0477 (17)	0.128 (4)	−0.0038 (16)	−0.001 (2)	0.005 (2)
C39	0.0669 (17)	0.0502 (16)	0.077 (2)	−0.0024 (13)	0.0069 (15)	0.0041 (15)

### Geometric parameters (Å, °)

**Table:**

Cu1—N1	2.0746 (18)	C17—C18	1.382 (4)
Cu1—P2	2.2643 (9)	C17—H17A	0.9300
Cu1—P1	2.2792 (8)	C18—C19	1.359 (4)
Cu1—Cl1	2.3831 (11)	C18—H18A	0.9300
P1—C16	1.826 (2)	C19—C20	1.362 (4)
P1—C10	1.829 (3)	C19—H19A	0.9300
P1—C4	1.832 (2)	C20—C21	1.390 (4)
P2—C22	1.822 (2)	C20—H20A	0.9300
P2—C34	1.824 (3)	C21—H21A	0.9300
P2—C28	1.832 (2)	C22—C23	1.376 (3)
N1—C3	1.316 (3)	C22—C27	1.395 (3)
N1—C1	1.355 (3)	C23—C24	1.379 (3)
N2—C3	1.324 (3)	C23—H23A	0.9300
N2—C2	1.346 (4)	C24—C25	1.365 (4)
N2—H2	0.80 (3)	C24—H24A	0.9300
C1—C2	1.351 (4)	C25—C26	1.371 (4)
C1—H1A	0.9300	C25—H25A	0.9300
C2—H2A	0.9300	C26—C27	1.394 (4)
C3—H3A	0.9300	C26—H26A	0.9300
C4—C5	1.364 (4)	C27—H27A	0.9300
C4—C9	1.375 (4)	C28—C33	1.389 (3)
C5—C6	1.392 (5)	C28—C29	1.390 (3)
C5—H5A	0.9300	C29—C30	1.383 (4)
C6—C7	1.351 (5)	C29—H29A	0.9300
C6—H6A	0.9300	C30—C31	1.363 (4)
C7—C8	1.349 (5)	C30—H30A	0.9300
C7—H7A	0.9300	C31—C32	1.373 (5)
C8—C9	1.380 (4)	C31—H31A	0.9300
C8—H8A	0.9300	C32—C33	1.383 (4)
C9—H9A	0.9300	C32—H32A	0.9300
C10—C15	1.375 (3)	C33—H33A	0.9300
C10—C11	1.385 (4)	C34—C35	1.390 (4)
C11—C12	1.379 (4)	C34—C39	1.394 (4)
C11—H11A	0.9300	C35—C36	1.394 (5)
C12—C13	1.365 (5)	C35—H35A	0.9300
C12—H12A	0.9300	C36—C37	1.374 (7)
C13—C14	1.361 (5)	C36—H36A	0.9300
C13—H13A	0.9300	C37—C38	1.363 (6)
C14—C15	1.392 (4)	C37—H37A	0.9300
C14—H14A	0.9300	C38—C39	1.370 (4)
C15—H15A	0.9300	C38—H38A	0.9300
C16—C17	1.381 (3)	C39—H39A	0.9300
C16—C21	1.388 (3)		
			
N1—Cu1—P2	105.54 (6)	C21—C16—P1	118.29 (18)
N1—Cu1—P1	106.78 (6)	C16—C17—C18	121.0 (3)
P2—Cu1—P1	123.54 (2)	C16—C17—H17A	119.5
N1—Cu1—Cl1	100.77 (6)	C18—C17—H17A	119.5
P2—Cu1—Cl1	109.34 (3)	C19—C18—C17	120.6 (3)
P1—Cu1—Cl1	108.45 (3)	C19—C18—H18A	119.7
C16—P1—C10	103.49 (10)	C17—C18—H18A	119.7
C16—P1—C4	103.02 (11)	C18—C19—C20	119.8 (3)
C10—P1—C4	101.45 (10)	C18—C19—H19A	120.1
C16—P1—Cu1	119.39 (8)	C20—C19—H19A	120.1
C10—P1—Cu1	114.03 (8)	C19—C20—C21	120.2 (3)
C4—P1—Cu1	113.32 (8)	C19—C20—H20A	119.9
C22—P2—C34	103.23 (11)	C21—C20—H20A	119.9
C22—P2—C28	101.52 (10)	C16—C21—C20	120.8 (2)
C34—P2—C28	104.71 (12)	C16—C21—H21A	119.6
C22—P2—Cu1	118.53 (8)	C20—C21—H21A	119.6
C34—P2—Cu1	111.89 (7)	C23—C22—C27	118.7 (2)
C28—P2—Cu1	115.29 (8)	C23—C22—P2	116.89 (17)
C3—N1—C1	104.9 (2)	C27—C22—P2	124.39 (18)
C3—N1—Cu1	128.80 (18)	C22—C23—C24	121.4 (2)
C1—N1—Cu1	126.30 (17)	C22—C23—H23A	119.3
C3—N2—C2	107.4 (2)	C24—C23—H23A	119.3
C3—N2—H2	125 (2)	C25—C24—C23	119.9 (3)
C2—N2—H2	128 (2)	C25—C24—H24A	120.1
C2—C1—N1	109.9 (3)	C23—C24—H24A	120.1
C2—C1—H1A	125.1	C24—C25—C26	120.2 (2)
N1—C1—H1A	125.1	C24—C25—H25A	119.9
N2—C2—C1	106.1 (3)	C26—C25—H25A	119.9
N2—C2—H2A	127.0	C25—C26—C27	120.5 (3)
C1—C2—H2A	127.0	C25—C26—H26A	119.8
N1—C3—N2	111.7 (3)	C27—C26—H26A	119.8
N1—C3—H3A	124.1	C26—C27—C22	119.5 (3)
N2—C3—H3A	124.1	C26—C27—H27A	120.3
C5—C4—C9	117.8 (2)	C22—C27—H27A	120.3
C5—C4—P1	118.6 (2)	C33—C28—C29	118.5 (2)
C9—C4—P1	123.61 (19)	C33—C28—P2	118.78 (18)
C4—C5—C6	120.1 (3)	C29—C28—P2	122.68 (19)
C4—C5—H5A	120.0	C30—C29—C28	120.4 (3)
C6—C5—H5A	120.0	C30—C29—H29A	119.8
C7—C6—C5	121.3 (3)	C28—C29—H29A	119.8
C7—C6—H6A	119.4	C31—C30—C29	120.6 (3)
C5—C6—H6A	119.4	C31—C30—H30A	119.7
C8—C7—C6	119.0 (3)	C29—C30—H30A	119.7
C8—C7—H7A	120.5	C30—C31—C32	119.7 (3)
C6—C7—H7A	120.5	C30—C31—H31A	120.1
C7—C8—C9	120.5 (3)	C32—C31—H31A	120.1
C7—C8—H8A	119.8	C31—C32—C33	120.6 (3)
C9—C8—H8A	119.8	C31—C32—H32A	119.7
C4—C9—C8	121.3 (3)	C33—C32—H32A	119.7
C4—C9—H9A	119.4	C32—C33—C28	120.2 (3)
C8—C9—H9A	119.4	C32—C33—H33A	119.9
C15—C10—C11	118.0 (3)	C28—C33—H33A	119.9
C15—C10—P1	124.9 (2)	C35—C34—C39	118.6 (3)
C11—C10—P1	117.12 (19)	C35—C34—P2	123.2 (2)
C12—C11—C10	121.6 (3)	C39—C34—P2	118.1 (2)
C12—C11—H11A	119.2	C34—C35—C36	118.8 (4)
C10—C11—H11A	119.2	C34—C35—H35A	120.6
C13—C12—C11	119.6 (3)	C36—C35—H35A	120.6
C13—C12—H12A	120.2	C37—C36—C35	120.9 (4)
C11—C12—H12A	120.2	C37—C36—H36A	119.5
C14—C13—C12	119.9 (3)	C35—C36—H36A	119.5
C14—C13—H13A	120.1	C38—C37—C36	120.5 (4)
C12—C13—H13A	120.1	C38—C37—H37A	119.7
C13—C14—C15	120.9 (3)	C36—C37—H37A	119.7
C13—C14—H14A	119.6	C37—C38—C39	119.2 (4)
C15—C14—H14A	119.6	C37—C38—H38A	120.4
C10—C15—C14	120.1 (3)	C39—C38—H38A	120.4
C10—C15—H15A	120.0	C38—C39—C34	121.9 (3)
C14—C15—H15A	120.0	C38—C39—H39A	119.0
C17—C16—C21	117.7 (2)	C34—C39—H39A	119.0
C17—C16—P1	123.99 (17)		
			
N1—Cu1—P1—C16	171.86 (10)	C13—C14—C15—C10	−0.4 (6)
P2—Cu1—P1—C16	−65.76 (9)	C10—P1—C16—C17	97.1 (3)
Cl1—Cu1—P1—C16	64.03 (9)	C4—P1—C16—C17	−8.2 (3)
N1—Cu1—P1—C10	−65.17 (10)	Cu1—P1—C16—C17	−134.9 (2)
P2—Cu1—P1—C10	57.21 (8)	C10—P1—C16—C21	−85.7 (2)
Cl1—Cu1—P1—C10	−173.00 (8)	C4—P1—C16—C21	168.98 (19)
N1—Cu1—P1—C4	50.22 (11)	Cu1—P1—C16—C21	42.3 (2)
P2—Cu1—P1—C4	172.60 (9)	C21—C16—C17—C18	−0.9 (5)
Cl1—Cu1—P1—C4	−57.61 (9)	P1—C16—C17—C18	176.3 (3)
N1—Cu1—P2—C22	175.02 (10)	C16—C17—C18—C19	0.8 (6)
P1—Cu1—P2—C22	52.08 (9)	C17—C18—C19—C20	−0.5 (5)
Cl1—Cu1—P2—C22	−77.34 (9)	C18—C19—C20—C21	0.3 (5)
N1—Cu1—P2—C34	55.07 (11)	C17—C16—C21—C20	0.7 (4)
P1—Cu1—P2—C34	−67.87 (10)	P1—C16—C21—C20	−176.7 (2)
Cl1—Cu1—P2—C34	162.71 (10)	C19—C20—C21—C16	−0.4 (4)
N1—Cu1—P2—C28	−64.44 (11)	C34—P2—C22—C23	161.39 (19)
P1—Cu1—P2—C28	172.63 (8)	C28—P2—C22—C23	−90.3 (2)
Cl1—Cu1—P2—C28	43.21 (9)	Cu1—P2—C22—C23	37.1 (2)
P2—Cu1—N1—C3	−41.3 (2)	C34—P2—C22—C27	−21.6 (2)
P1—Cu1—N1—C3	91.7 (2)	C28—P2—C22—C27	86.7 (2)
Cl1—Cu1—N1—C3	−155.1 (2)	Cu1—P2—C22—C27	−145.94 (19)
P2—Cu1—N1—C1	140.4 (2)	C27—C22—C23—C24	0.5 (4)
P1—Cu1—N1—C1	−86.6 (2)	P2—C22—C23—C24	177.6 (2)
Cl1—Cu1—N1—C1	26.6 (2)	C22—C23—C24—C25	−0.1 (4)
C3—N1—C1—C2	−1.3 (4)	C23—C24—C25—C26	−0.4 (5)
Cu1—N1—C1—C2	177.4 (2)	C24—C25—C26—C27	0.6 (5)
C3—N2—C2—C1	−0.3 (4)	C25—C26—C27—C22	−0.3 (4)
N1—C1—C2—N2	1.0 (4)	C23—C22—C27—C26	−0.3 (4)
C1—N1—C3—N2	1.1 (3)	P2—C22—C27—C26	−177.2 (2)
Cu1—N1—C3—N2	−177.48 (18)	C22—P2—C28—C33	144.4 (2)
C2—N2—C3—N1	−0.6 (4)	C34—P2—C28—C33	−108.5 (2)
C16—P1—C4—C5	−91.1 (3)	Cu1—P2—C28—C33	14.9 (2)
C10—P1—C4—C5	162.0 (3)	C22—P2—C28—C29	−32.6 (2)
Cu1—P1—C4—C5	39.4 (3)	C34—P2—C28—C29	74.6 (2)
C16—P1—C4—C9	89.2 (3)	Cu1—P2—C28—C29	−162.0 (2)
C10—P1—C4—C9	−17.7 (3)	C33—C28—C29—C30	2.0 (4)
Cu1—P1—C4—C9	−140.3 (2)	P2—C28—C29—C30	178.9 (2)
C9—C4—C5—C6	−3.6 (7)	C28—C29—C30—C31	−0.9 (5)
P1—C4—C5—C6	176.7 (4)	C29—C30—C31—C32	−0.5 (5)
C4—C5—C6—C7	3.1 (9)	C30—C31—C32—C33	0.9 (5)
C5—C6—C7—C8	−0.5 (9)	C31—C32—C33—C28	0.2 (5)
C6—C7—C8—C9	−1.5 (7)	C29—C28—C33—C32	−1.6 (4)
C5—C4—C9—C8	1.6 (5)	P2—C28—C33—C32	−178.7 (2)
P1—C4—C9—C8	−178.7 (3)	C22—P2—C34—C35	121.6 (2)
C7—C8—C9—C4	1.0 (6)	C28—P2—C34—C35	15.7 (3)
C16—P1—C10—C15	0.5 (3)	Cu1—P2—C34—C35	−109.8 (2)
C4—P1—C10—C15	107.1 (2)	C22—P2—C34—C39	−62.6 (2)
Cu1—P1—C10—C15	−130.7 (2)	C28—P2—C34—C39	−168.5 (2)
C16—P1—C10—C11	−178.6 (2)	Cu1—P2—C34—C39	66.0 (2)
C4—P1—C10—C11	−72.0 (2)	C39—C34—C35—C36	2.3 (5)
Cu1—P1—C10—C11	50.2 (2)	P2—C34—C35—C36	178.0 (3)
C15—C10—C11—C12	−2.3 (4)	C34—C35—C36—C37	0.0 (6)
P1—C10—C11—C12	176.9 (2)	C35—C36—C37—C38	−1.9 (7)
C10—C11—C12—C13	1.5 (5)	C36—C37—C38—C39	1.5 (6)
C11—C12—C13—C14	0.0 (5)	C37—C38—C39—C34	0.8 (5)
C12—C13—C14—C15	−0.5 (6)	C35—C34—C39—C38	−2.7 (4)
C11—C10—C15—C14	1.7 (4)	P2—C34—C39—C38	−178.7 (2)
P1—C10—C15—C14	−177.4 (3)		

### Hydrogen-bond geometry (Å, °)

**Table:**

*D*—H···*A*	*D*—H	H···*A*	*D*···*A*	*D*—H···*A*
N2—H2···Cl1^i^	0.80 (4)	2.34 (4)	3.127 (3)	171 (3)
C5—H5A···Cl1	0.93	2.78	3.663 (4)	160

Symmetry codes: (i) −*x*+3/2, *y*−1/2, −*z*+3/2.
